# Inverse design mechanical metamaterials with dual load-bearing and heat-transfer capabilities

**DOI:** 10.1371/journal.pone.0345912

**Published:** 2026-04-06

**Authors:** Yuhao Liu

**Affiliations:** State Key Laboratory of Structural Analysis, Optimization and CAE Software for Industrial Equipment, Dalian University of Technology, Dalian, China; University of Vigo, SPAIN

## Abstract

Metamaterials can achieve extraordinary properties unattainable in natural materials through sophisticated artificial structural design. This study constructs novel tri-periodic minimal surface configurations based on fundamental structures. By employing surface boundary capture techniques to capture infinitely continuous and smooth surfaces, corresponding fusion functions are derived. New configurations are then established: one fused with rod elements and another featuring smooth fusion of multiple fundamental structures. By integrating the advantages of these configurations, novel metamaterials with both excellent load-bearing and heat transfer properties can be designed. Transition fusion functions are employed to derive implicit function construction methods for each new configuration. Through homogenisation analysis of all novel configurations, this study obtains their mechanical vector data. Combined with the implicit function expressions, this configuration and design methodology establishes a novel theoretical approach for reverse-engineering mechanical metamaterials according to specific requirements.

## 1. Introduction

With the advancement of aerospace, high-speed vehicles, unmanned systems, and advanced energy equipment, engineering structures now face multiple performance demands simultaneously, including lightweighting, high load-bearing capacity, and efficient thermal management [1–3]. Traditional material systems primarily enhance performance through composition design or microstructural regulation; however, against the backdrop of escalating multifunctional integration requirements, their developmental potential has gradually approached its limits [4–6]. Consequently, metamaterials—which achieve quantum leaps in material properties through geometric and topological design—have emerged as a pivotal research frontier in structural and materials science in recent years [7–9]. Traditional approaches to developing novel metamaterials typically involve precision processing [10–14] and composition manipulation (such as alloying elements in metals) [15–19].

Porous structures exhibit significant advantages in load-bearing structures, energy absorption, and thermal management systems due to their low density, high specific surface area, and excellent internal connectivity [20–21]. Among these, Triply Periodic Minimal Surface (TPMS) structures are widely regarded as the most promising class of porous metamaterials for engineering applications, due to their inherent geometric continuity, smooth curvature, and periodic symmetry [22–26]. Extensive research indicates that different TPMS fundamental configurations (such as Gyroid, Diamond, I-WP, etc.) exhibit significant variations in equivalent stiffness, deformation coordination, and thermal conduction pathway connectivity, making them important candidates for integrated structure-function design [27–29].

Regarding the performance regulation of TPMS structures, existing research has proposed multiple design strategies, including relative density modulation, wall thickness gradient design, multi-scale lattice nesting, and parametric design methods based on numerical optimisation. For instance, a reversible design framework based on implicit function fusion achieved synergistic optimisation of dual load-bearing and heat transfer functions, significantly enhancing structural inverse design capabilities. The application of homogenisation methods and equivalent solid models reduced simulation computation time by over 99%, providing a viable pathway for rapid analysis of gradient TPMS structures. Topology optimisation strategies incorporating artificial neural networks (ANNs) achieved precise control of nonlinear gradients through voxel modelling, thereby enhancing the automation of multiscale design. Strain energy-driven multi-topology fusion methods, via interface interpolation optimisation, increased structural stiffness by over 20% [30–33]. Nevertheless, existing approaches exhibit notable limitations, including inadequate assurance of geometric continuity for complex transitional boundaries, diminished predictive accuracy of models during plastic deformation stages, insufficient validation of performance deviations caused by manufacturing defects, and insufficient consideration of multiphysics coupling in design. While these approaches have achieved progress in specific mechanical or thermal metrics, they generally remain within the forward design paradigm. This involves obtaining performance improvements through parameter scans or optimisation iterations within a predetermined structural form, making it difficult to directly achieve structural back-design starting from target performance.

In recent years, researchers have focused on exploring the synergistic optimisation of structural design and additive manufacturing processes based on triple-periodic minimal surfaces (TPMS). For instance, a recent study published in Additive Manufacturing (2024, 95:104544) systematically analysed the coupling effects between printing parameters (such as temperature, layer height, and speed) and four TPMS topologies (Gyroid, Neovius, Diamond, I-WP) to achieve tunable control of the X-band electromagnetic interference shielding effectiveness (SET) of PVDF-graphene composites. The Neovius structure demonstrated a shielding effectiveness of up to 75 dB under specific orientations, significantly outperforming conventional solid structures [34]. Meanwhile, a study in Thin-Walled Structures (2025, 215:113442) further explored the influence of hybrid ratios (R_{HB}) on energy absorption characteristics and impact resistance by introducing multi-phase TPMS lattice designs (e.g., hybrids of I-WP and Gyroid). It was discovered that the suppression of crack propagation by non-uniform phase boundaries enabled the structure to exhibit supernormal specific energy absorption (SEA reaching 40.42 J/g) under both quasi-static and dynamic loading [35]. These studies conclusively demonstrate that through multi-topology fusion and synergistic process parameter design, the functional performance boundaries of structures can be significantly expanded without altering the intrinsic composition of the material.

Beyond traditional engineering applications such as load-bearing structures, energy absorption, and thermal management, TPMS and its composite structures have also demonstrated broad application prospects in emerging functional materials in recent years. For instance, in soft actuators and flexible actuation systems, research indicates that introducing periodic or quasi-periodic structures with directional stiffness characteristics enables effective regulation of deformation modes and output forces while maintaining overall suppleness. This significantly enhances drive efficiency and controllability. Relevant work has been validated through numerical simulations and experiments, demonstrating the efficacy of structural topological design in regulating the anisotropic mechanical response of composite materials [36]. Furthermore, porous elastomers based on TPMS structures have garnered attention in the fields of tissue engineering scaffolds and smart materials. Existing research has conducted systematic numerical analyses of dielectric elastomers featuring TPMS topologies. Results indicate that different surface configurations and spatial orientations significantly influence the deformation capacity and stability of materials during electromechanically coupled actuation. Such studies further demonstrate that TPMS structures can not only serve as load-bearing or energy-absorbing units but also enable the regulation of multi-physics response behaviours through topological design, thereby expanding their application boundaries within functional metamaterials [37].

However, existing hybrid TPMS or lattice structure design methodologies still exhibit several critical shortcomings. Firstly, most research focuses on continuous construction at the geometric level, lacking a unified mathematical description framework; transitions between different topologies often rely on empirical geometric operations. Secondly, the relationship between structural parameters and macroscopic mechanical or thermal performance is predominantly presented in qualitative or case-study formats, with no established quantitative mapping for engineering design. More critically, these methods generally struggle to support inverse design for targeted performance requirements—that is, they cannot directly reverse-engineer structural topology and key parameters from anticipated load-bearing or heat-transfer capabilities.

Against this research backdrop, this paper takes the fundamental configuration of a typical TPMS as its subject. By incorporating surface boundary capture and implicit function modelling concepts, it establishes a unified description framework for TPMS based on transition blending functions. This approach achieves smooth transitions between different TPMS configurations within the implicit function space, fundamentally eliminating the geometric discontinuities and stress concentration issues commonly encountered in traditional piecemeal designs. Building upon this foundation, this paper systematically designs and analyses multiple novel hybrid TPMS structural forms, including rod-element reinforced structures and multi-TPMS continuous transition fusion structures.

This paper employs homogenisation theory to systematically compute the equivalent elastic stiffness matrix and thermal transmission response of the aforementioned structure, revealing in vectorised form the intrinsic relationship between transition parameters, topological configurations, and macroscopic performance. Integrated within an implicit modelling framework, these results enable the selection of structural topology and parameter tuning to directly serve specified performance requirements, thereby establishing a reverse design methodology for TPMS metamaterials oriented towards load-bearing and heat transfer co-optimisation. It should be noted that the thermal analysis herein focuses on heat conduction within the solid framework. Issues pertaining to flow within the pores, including convective heat transfer, permeability, and pressure drop, are not addressed in this paper.

The principal innovations and contributions of this paper may be summarised as follows:

(1) Direct embedding of transition blending functions within the implicit function modelling framework of TPMS, enabling continuous and differentiable fusion between diverse topological structures whilst avoiding geometric discontinuities;
(2) Establishing a quantitative mapping relationship between transition parameters and the homogenised equivalent elastic stiffness matrix, thereby revealing the connection between structural parameters and macroscopic performance;
(3) Proposing a structural design framework supporting inverse design, enabling the selection of suitable TPMS topology types and transition parameters based on target mechanical–thermal performance ranges.

## 2. Design models and theories

### 2.1. Design models

**Fig 1:**
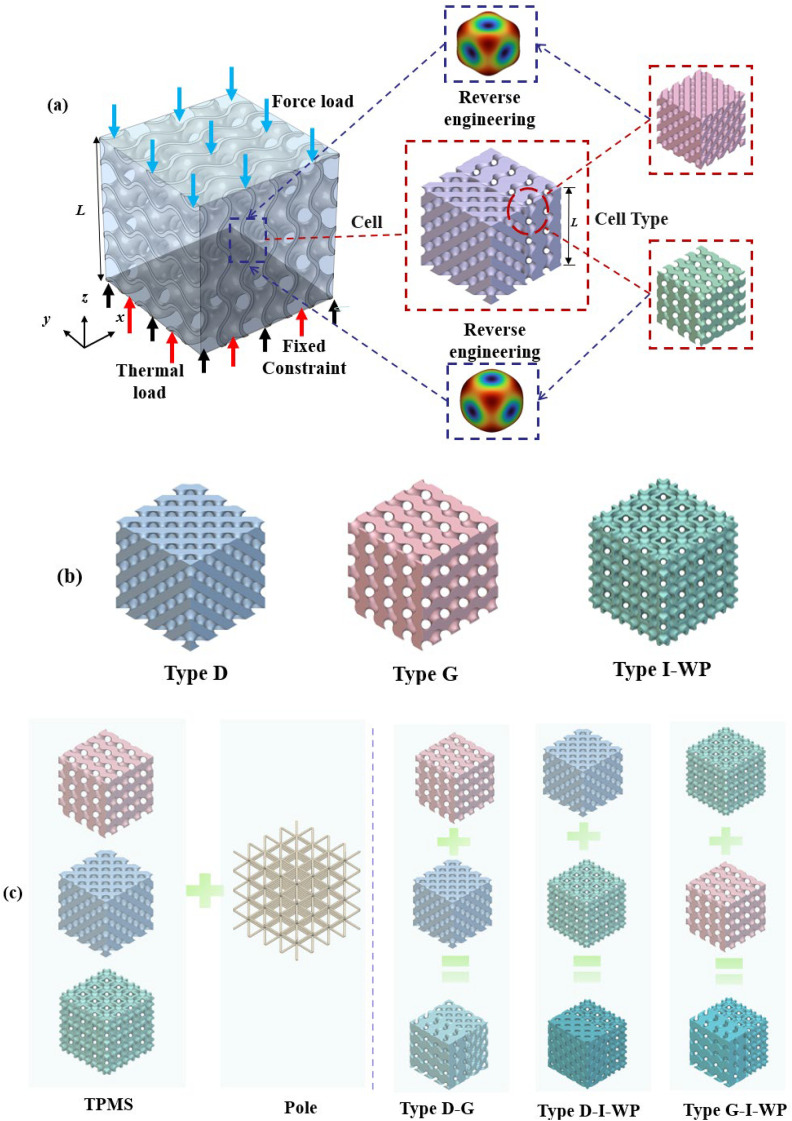
Design Model and Research Approach. Fig 1 shows the fusion design of the three-periodic minimal surface in various configurations.

Within the research framework illustrated in Fig 1, this paper employs a single-cell structure as the fundamental subject of study. A fixed constraint is applied to its base surface to establish fixed boundary conditions, with mechanical and thermal loads applied along the z-direction, respectively. The mechanical load is maintained at a constant magnitude of 1000 N; the thermal boundary condition is set to a constant temperature of 373.15 K at the base surface, while the remaining surfaces employ convective heat transfer boundaries with a heat transfer coefficient of 1500 W·m ⁻ ²·K ⁻ ¹. The interfacial thermal resistance is taken as 1 × 10 ⁻ ¹⁰ m²·K·W ⁻ ¹, the surface emissivity is 0.1, and the initial temperature is 273.15 K. Under unified force-heat coupling conditions, the system designed and analysed nine distinct TPMS unit cell configurations. These encompassed three fundamental configurations—Diamond (D-type), Gyroid (G-type), and I-WP-type—alongside enhanced structures formed by integrating these configurations with rod elements. Additionally, three continuous transition hybrid configurations were examined: D–G, D–I-WP, and G–I-WP. The figure schematically illustrates the D–G transition fusion unit cell. The remaining configurations and their corresponding mechanical and thermal performance characteristics will be systematically presented in subsequent sections.

Building upon this foundation, this paper directly embeds the transition fusion function into the implicit function modelling framework of TPMS. This enables continuous, differentiable transitions between multiple topological configurations under a unified mathematical description, thereby avoiding the interface discontinuities and abrupt property changes commonly encountered in traditional geometric splicing methods. Through homogenisation analysis of various configurations, equivalent elastic properties are derived, and the influence of different topological parameters on macroscopic mechanical responses is characterised in vectorised form. Combined with implicit function editing formulas, these results enable direct mapping of structural topology types and key transition parameters to target mechanical–thermal performance ranges. This establishes a performance-driven inverse design pathway for metamaterials, providing a feasible and universally applicable design methodology for the engineering application of multifunctional TPMS metamaterials in synergistic optimisation of load-bearing and thermal management.

As shown in Table 1, the model material employed in this study is aluminium alloy 6061-T6. As a typical precipitation-hardened aluminium alloy, this material combines excellent mechanical properties with favourable physical characteristics. Its Young’s modulus is approximately 68.9 GPa, while its shear modulus is around 26.0 GPa, exhibiting high rigidity and stable elastic response. This satisfies the load-bearing and deformation control requirements for lightweight structural components. Concurrently, this material achieves a yield strength of 276 MPa and a tensile strength of approximately 310 MPa, demonstrating commendable strength levels and fatigue resistance. These attributes confer significant advantages in high-strength, lightweight design applications.

**Table 1:** Aluminium 6061-T6 Material Specifications.

Young’s modulus	68.9GPa
Shear modulus	26.0GPa
Poisson ratio	0.33
Yield strength	276MPa
Tensile strength	310MPa
Elongation	12%
Density	2.70g/cm^3
Thermal conductivity	167W/(m · K)
Specific heat capacity	896J/(kg · K)
Electrical conductivity	40%IACS
Melting point	855.15K-925.15K

Detailed Specification Table for Aluminum Alloy 6061-T6 Material.

In terms of physical properties, the 6061-T6 aluminium alloy boasts a density of merely 2.70 g/cm³, significantly lower than conventional steels, conferring superior specific strength and specific stiffness. Its thermal conductivity of approximately 167 W/(m·K) ensures excellent heat transfer capabilities, rendering it suitable for heat exchange and thermal management structures. Furthermore, with a specific heat capacity of 896 J/(kg·K), this material also holds potential for thermal protection and energy absorption applications.

### 2.2. Formula for the integration function

**Fig 2:**
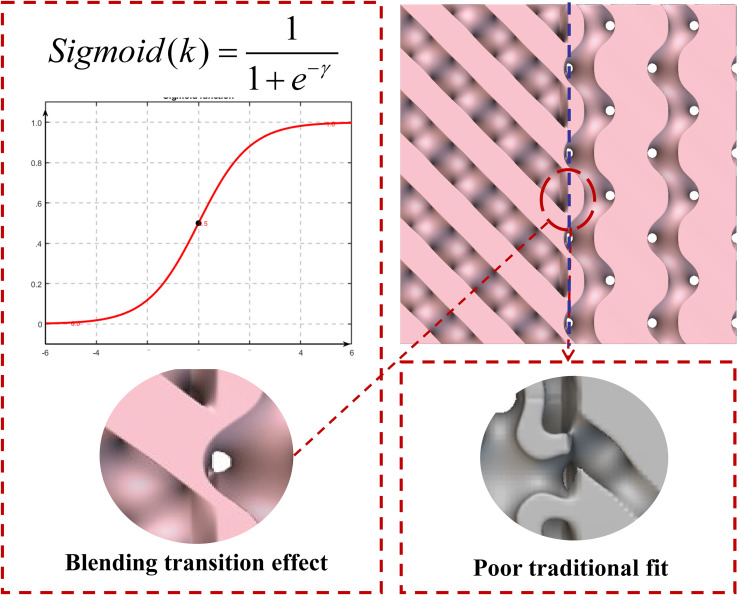
Schematic of the Fusion Transition Function. Fig 2 shows the detailed method illustration of the smooth transition fusion function.

As illustrated in Fig 2, traditional modelling software such as Rhino, SolidWorks, and UG NX can achieve the combination of unit cells. However, they cannot capture surfaces according to surface distribution patterns. Forced combinations result in discontinuities as shown, leading to failures in structural mechanical performance. Their capabilities are insufficient for the effective fusion of multi-type unit cells. This research employs a method incorporating implicit functions with transitional fusion, combined with custom-programmed efficient code, to resolve this issue and achieve seamless integration of crystal cells.

The sigmoid function, also known as the S-shaped function, is a class of nonlinear mapping functions widely employed in mathematics and engineering. Its typical form is $Sigmoid\left(k\right)=\frac{\text{1}}{1+e^{-\gamma }}$. This function maps any real number input to the interval (0,1), exhibiting a smooth S-shaped curve characteristic. As x approaches negative infinity, the function value approaches zero; as x approaches positive infinity, the function value approaches one; and at x = 0, it exhibits symmetry with a function value of 0.5. From a mathematical perspective, the sigmoid function is continuous and differentiable, with its first derivative possessing an analytical form [38–39]:


$$Sigmoid^{\prime }\left(k\right)\text{=}Sigmoid\left(k\right)\text{(1-}Sigmoid\left(k\right))$$ (1)

This self-referential derivative property lends it considerable utility in numerical computation and optimisation processes. Owing to its monotonically increasing nature and constrained output range, the sigmoid function is frequently employed in probabilistic modelling and binary classification tasks, where it is interpreted as an “activation function” capable of performing non-linear mapping and normalisation of input signals [40]. Within neural networks, it has been extensively adopted to introduce nonlinear elements, enabling models to approximate complex functions [41].

By leveraging the properties of this function, we can utilise its monotonicity and smoothness to capture the distribution of isovalue points on minimal surfaces as transition blending functions. Combined with the implicit function formula discussed in the next chapter, we can directly obtain polycrystalline structures that realise transition blending through structural optimisation and self-programming code.

The transitional fusion TPMS structure investigated herein differs geometrically from strictly periodic lattice configurations. As distinct TPMS fundamental configurations evolve continuously through space via the transitional fusion function, a degree of geometric inhomogeneity manifests within the unit cell along specific directions. Consequently, classical homogenisation theory based on the assumption of perfect periodicity requires explicit clarification of its applicability conditions when employed.

From the perspective of scale separation, the transition-blended structure employed in this study still satisfies the fundamental conditions for homogenisation analysis. Despite the presence of geometric gradients within the unit cell, its characteristic lengths (such as minimum wall thickness, surface curvature radius, and transition zone length) are significantly smaller than the macroscopic scale of the overall structure. Consequently, the selected representative volume elements retain statistical representativeness at the spatial scale. Concurrently, the transition blending function maintains continuity and differentiability within the implicit function space. This ensures smooth transitions in both geometric and material distributions, thereby avoiding local singular responses caused by sharp discontinuous interfaces. Consequently, the equivalent field quantities possess a well-defined average meaning within the unit cell scale.

Given these characteristics, the homogenisation analysis herein is not employed to obtain strictly periodic limit solutions, but rather to characterise the evolution of effective mechanical and thermal properties driven by varying topological fusion methods and transition parameters. This approach is widely adopted in studies of gradient materials and non-perfectly periodic metamaterials, offering the advantage of maintaining computational efficiency while providing a consistent equivalent evaluation basis for structural scheme comparison and selection.

During numerical implementation, all configurations employ uniform unit cell dimensions, boundary conditions, and loading methods, ensuring comparability of homogenisation results across different design schemes. Although transitional fused structures do not satisfy strict periodicity assumptions, their equivalent performance results remain valid for assessing relative differences in macroscopic properties among various topological designs, thereby providing a sound basis for subsequent inverse design and engineering applications.

### 2.3. Implicit function formula and effective elasticity matrix

In TPMS structural research, an implicit function denotes an equation representation that defines surface f(x,y,z) using a continuous three-dimensional scalar function. Implicit functions not only provide a parametric and analytical geometric description but also enable flexible control over the solid and void regions of the structure by adjusting the level set threshold. This facilitates tunable porosity, specific surface area, and mechanical properties. Consequently, implicit functions serve as the core tool for TPMS mathematical modelling and numerical simulation, forming the foundation for structural optimisation and multiphysics simulation [42–43].

To achieve effective fusion of different types of unit cells, the latent function of the fundamental configuration unit cell must first be determined. Previous research [44–45] has established that the latent function for the D-type unit cell is:


$$\phi_{D}\left(\gamma \right)=cos\left(X\right)cos\left(Y\right)cos\left(Z\right)-sin\left(X\right)sin\left(Y\right)sin\left(Z\right)=C$$ (2)

The implicit function for the G-type unit cell is:


$$\phi_{G}\left(\gamma \right)=sin\left(X\right)cos\left(Y\right)+sin\left(Z\right)cos\left(X\right)+sin\left(Y\right)cos\left(Z\right)=C$$ (3)

The implicit function for the D-type unit cell is:


$$\begin{array}{c} \phi_{I-WP}\left(\gamma \right)=2\left[cos\left(X\right)cos\left(Y\right)+cos\left(Y\right)cos\left(Z\right)+cos\left(Z\right)cos\left(X\right)\right]\\ -\left[cos\left(2X\right)+cos\left(2Y\right)+cos\left(2Z\right)\right]=C \end{array}$$ (4)

Combining the sigmoid function, we first take the example of a transition fusion between G-type and D-type unit cells. Defining a smooth transition from G-type to D-type along the z-direction, the basic configuration of the fusion function is:


$$\phi_{\text{fusion}}\left(\gamma \right)=w\left(\gamma \right)\cdot \phi_{G}\left(\gamma \right)+\left[1-w\left(\gamma \right)\right]\cdot \phi_{D}\left(\gamma \right)$$ (5)

By substituting both unit configurations into the equation, the G-D type transition fusion latent function formula can be derived:


$$\begin{array}{c} \begin{array}{cc} \phi_{\text{fusion}}\left(\gamma \right) & =\frac{\text{1}}{1+e^{-k(\gamma -\gamma_{\text{0}})}}\cdot \left[sin\left(X\right)cos\left(Y\right)+sin\left(Z\right)cos\left(X\right)+sin\left(Y\right)cos\left(Z\right)\right]\\ & \begin{array}{c} \text{\textbackslash{}hspace\{0.33em}\;\;\;\}+\\ \frac{e^{-k(\gamma -\gamma_{\text{0}})}}{1+e^{-k(\gamma -\gamma_{\text{0}})}}\cdot \left[cos\left(X\right)cos\left(Y\right)cos\left(Z\right)-sin\left(X\right)sin\left(Y\right)sin\left(Z\right)\right] \end{array} \end{array} \end{array}$$ (6)

All transition parameters are adjustable. Taking the centre point as an example in this study, the parameter meanings are customised and named as shown in Table 2. Based on Table 2, the function can be modified to control the various parameters of the crystal cell after transition fusion.

**Table 2:** Implicit function control parameters.

Parameters	Physical significance	Value range	Effect
γ	Transition control variable	(−∞,+∞)	Control the degree of fusion
$\gamma_{\text{0}}$	Transition Centre Point	Real number	Determine the 50% fusion position
k	Transition Steepness Coefficient	*k* > 0	Control the smoothness of transitions
ω(γ)	Weighting function	(0,1)	Weighting coefficient for Type G
1−ω(γ)	Complementary weighting	(0,1)	Weighting coefficient for Type D

Detailed Description Table of Implicit Function Control Parameters.

Similarly, the functions for the G-I-WP type fusion crystal cell (50% fusion position) can be derived as follows:


$$\begin{array}{c} \begin{array}{cc} \phi_{\text{fusion}}\left(\gamma \right) & =\frac{\text{1}}{1+e^{-k(\gamma -\gamma_{\text{0}})}}\cdot \left[sin\left(X\right)cos\left(Y\right)+sin\left(Z\right)cos\left(X\right)+sin\left(Y\right)cos\left(Z\right)\right]\\ & \text{\textbackslash{}hspace\{1em}\}+\frac{e^{-k(\gamma -\gamma_{\text{0}})}}{1+e^{-k(\gamma -\gamma_{\text{0}})}}\cdot [2\left[cos\left(X\right)cos\left(Y)+cos(Y\right)cos\left(Z)+cos(Z\right)cos\left(X\right)\right]\\ & \text{\textbackslash{}hspace\{1em}\}-\left[cos\left(2X)+cos(2Y)+cos(2Z\right)\right]] \end{array} \end{array}$$ (7)

The implicit function for the D-I-WP fusion crystal cell (50% fusion position) is:


$$\begin{array}{c} \begin{array}{cc} \phi_{\text{fusion}}\left(\gamma \right) & =\frac{\text{1}}{1+e^{-k(\gamma -\gamma_{\text{0}})}}\cdot \left[cos\left(X\right)cos\left(Y\right)cos\left(Z\right)-sin\left(X\right)sin\left(Y\right)sin\left(Z\right)\right]\\ & \text{\textbackslash{}hspace\{1em}\}+\frac{e^{-k(\gamma -\gamma_{\text{0}})}}{1+e^{-k(\gamma -\gamma_{\text{0}})}}\cdot [2\left[cos\left(X\right)cos\left(Y)+cos(Y\right)cos\left(Z)+cos(Z\right)cos\left(X\right)\right]\\ & \text{\textbackslash{}hspace\{1em}\}-\left[cos\left(2X)+cos(2Y)+cos(2Z\right)\right]] \end{array} \end{array}$$ (8)

Furthermore, in Chapter 3, we calculated the homogenised elastic stiffness matrix for various cell types. This serves as an effective constitutive tensor describing the equivalent elastic response of periodic or heterogeneous structures at the macroscale. The stiffness matrix characterises the macroscopically equivalent mechanical properties of microscopically complex structures such as TPMS and porous materials. It “averages” the influence of microstructures on the overall elastic response, thereby enabling complex materials to be equivalently represented as homogeneous quasi-isotropic continua [46–47] for quantifying their mechanical properties, as shown in Equation (9).


$$\begin{array}{c} \left[C\right]=\left[\begin{array}{cccccc} @cccccc@C_{11} & C_{12} & C_{13} & C_{14} & C_{15} & C_{16}\\ C_{21} & C_{22} & C_{23} & C_{24} & C_{25} & C_{26}\\ C_{31} & C_{32} & C_{33} & C_{34} & C_{35} & C_{36}\\ C_{41} & C_{42} & C_{43} & C_{44} & C_{45} & C_{46}\\ C_{51} & C_{52} & C_{53} & C_{54} & C_{55} & C_{56}\\ C_{61} & C_{62} & C_{63} & C_{64} & C_{65} & C_{66} \end{array}\right] \end{array}$$ (9)

In the aforementioned matrix, $C_{11}$, $C_{12}$, $C_{13}$ reflects the equivalent Young’s modulus in the principal direction, $C_{44}$, $C_{55}$, $C_{66}$ corresponds to the equivalent shear modulus in different directions, while the off-diagonal elements reveal the coupling relationship between the Poisson effect under anisotropy and the principal direction.

### 2.4. Numerical implementation and convergence

**Fig 3:**
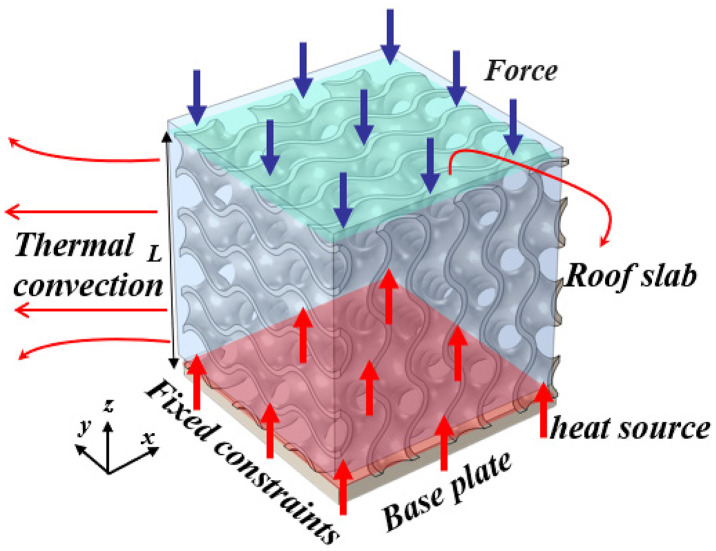
Boundary conditions. Fig 3 shows the application of boundary conditions for a three-period minimal surface unit cell in the numerical simulation.

As illustrated in Fig 3, this paper conducts a force-thermal coupled numerical analysis on the TPMS unit cell structure. Mechanical and thermal boundary conditions are uniformly applied to the same geometric model to ensure consistency and comparability in the multiphysics analysis. In the mechanical analysis, the cell base was defined as a fixed constraint boundary, with all three translational degrees of freedom restrained to simulate the rigid connection between the structure and its substrate or support layer under macro-scale loading conditions. A constant normal force was applied along the z-direction to the cell top surface, representing the overall mechanical response of the structure under axial compression.

In thermal analysis, the unit cell base simultaneously serves as a heat source boundary, with a constant temperature condition applied to simulate the thermal input region. All other external surfaces employ convective heat transfer boundary conditions to describe the thermal exchange process between the structure and its external environment. A fixed convective heat transfer coefficient is adopted to reflect heat dissipation characteristics under forced convection conditions. Concurrently, the thermal-structural coupling analysis incorporates interfacial thermal resistance and surface radiation effects to ensure the completeness of the heat transfer pathway description. The configuration of these boundary conditions establishes a clearly defined, physically closed system for the structure concerning thermal input, thermal diffusion, and mechanical constraints. This enables a relatively realistic representation of the service conditions encountered by TPMS metamaterials in engineering applications.

Given that the TPMS structure is defined by implicit surfaces, exhibiting highly variable curvature and complex connectivity, the computational domain is discretised using an unstructured tetrahedral mesh. Compared to structured meshes, unstructured tetrahedral grids exhibit superior geometric fitting capabilities over complex curved surfaces. This effectively mitigates mesh distortion in regions of high local curvature, thereby enhancing the stability and accuracy of numerical computations.

During mesh generation, element sizing adheres to a ‘curvature-adaptive’ principle: locally refined meshes are applied over high-curvature TPMS surfaces and transitional blending zones to accurately capture geometric details and stress–temperature gradient variations. Conversely, coarser element sizes are employed in regions with relatively smooth curvature variations to control overall computational scale. This approach effectively reduces computational cost while maintaining numerical accuracy. Furthermore, identical mesh generation strategies and quality control standards were applied across all cases to ensure robust comparability of numerical results between different configurations.

**Fig 4:**
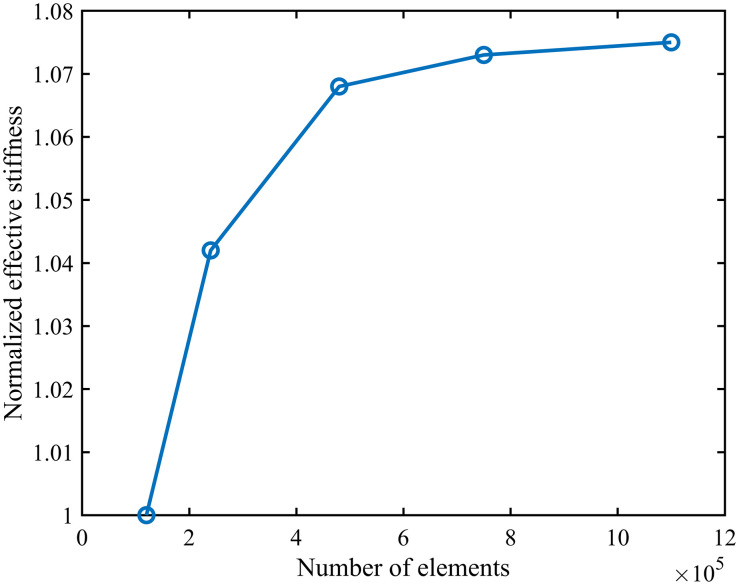
Grid independence verification. Fig 4 shows the independent verification diagram conducted in the numerical simulation to ensure the accuracy of the simulation and to enhance the efficiency of the numerical simulation.

To validate the sensitivity of numerical results to mesh discretisation, a systematic mesh independence analysis was conducted on the selected configuration, as illustrated in Fig 4 above. By progressively refining the unstructured mesh and comparing variations in key response quantities (such as equivalent elastic modulus or maximum equivalent stress) across different mesh densities, the convergence of numerical results with increasing mesh refinement could be assessed.

Results indicate that once the number of elements reaches a certain threshold, the variation in key mechanical response quantities diminishes significantly. Further refinement of the mesh has a negligible impact on computational outcomes. Balancing accuracy with computational efficiency, the mesh scheme corresponding to a response quantity variation rate below the preset tolerance range is selected as the unified mesh configuration for subsequent calculations. The aforementioned findings demonstrate that the mesh partitioning strategy employed in this study ensures both computational accuracy and the reliability and reproducibility of numerical results.

## 3. Results and analysis

As shown in Table 3 above, this presents a schematic modelling of a typical TPMS unit cell and its 5 × 5 lattice. Different configurations exhibit variations in geometric characteristics and topological complexity, thereby demonstrating distinct behaviours in the regulation of mechanical and thermal properties. The Diamond (Type D) unit cell exhibits strong isotropy and load-bearing capacity, making it suitable for applications requiring both strength and thermal conductivity. The Gyroid (Type G) structure, characterised by continuous double-helix channels, possesses excellent permeability and specific surface area, rendering it ideal for scenarios involving enhanced heat transfer and flow coupling. The I-WP configuration exhibits high pore connectivity, though local curvature discontinuities may induce stress concentration, achieving a balance between thermal conductivity and specific strength. Pole structures, while not typical TPMS, feature clear load transfer pathways and straightforward fabrication, originating from the addition of fused supports within complex unit cells. Fused transition unit cells achieve multi-property regulation through the fusion of different configurations. Type D-G combines the mechanical stability of Type D with the high connectivity of Type G. Type G-I-WP integrates the continuous channels of Type G with the local complexity of I-WP to enhance specific surface area. Type D-I-WP, meanwhile, establishes coordination between the isotropy of Type D and the topological complexity of I-WP.

**Table 3:** Schematic modelling of various crystal cell types.

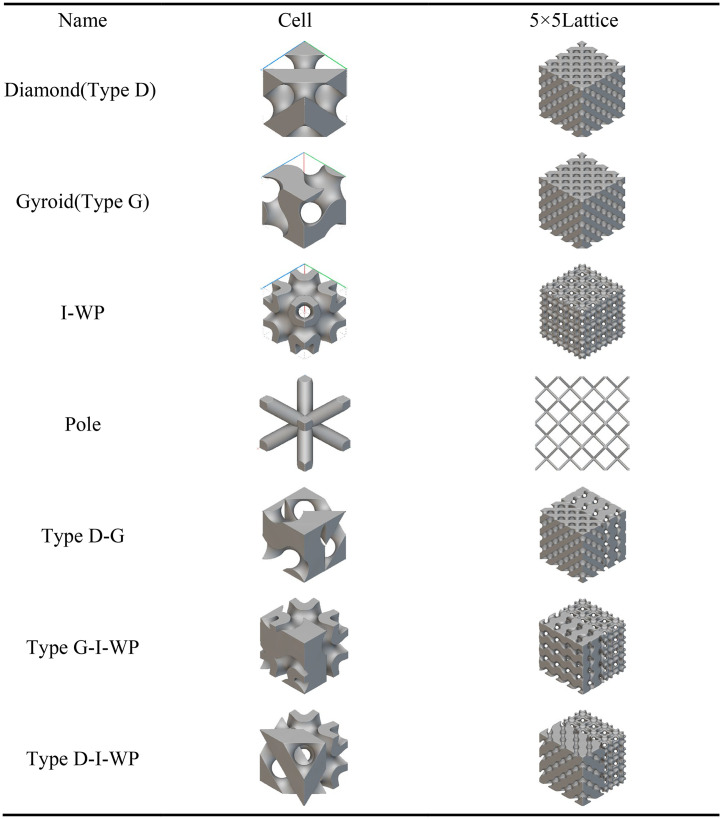

### 3.1. Static analysis

Table 4 presents the mechanical responses of conventional, G-type, and I-WP-type TPMS crystalline cells under static loading. The computational model employs a bottom-fixed constraint with a uniform vertical load of 1000 N applied at the top. Results are displayed as cross-sectional and overall contour plots. The Figs reveal significant differences in stress distribution and deformation patterns among the three cell types.

**Table 4:** Static analysis of traditional unit cells.

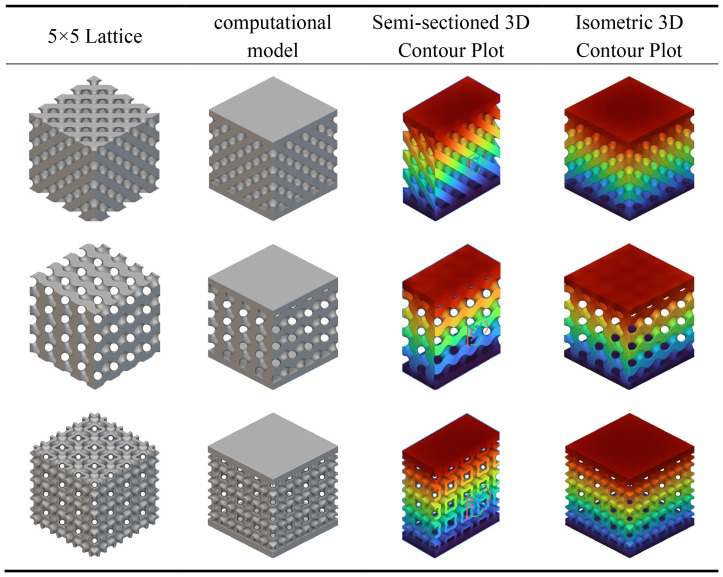

The D-type unit cell, owing to its quasi-isotropic characteristics, transmits loads relatively uniformly throughout the space, resulting in minimal overall deformation and demonstrating excellent load-bearing stability. The G-type unit cell features continuous helical channels; under load, stresses are primarily distributed along the channel walls. Although local deformation is slightly higher, its strong connectivity provides superior energy dissipation and structural toughness. In contrast, the I-WP unit cell exhibits greater geometric complexity, leading to more pronounced localised stress concentration under loading. Whilst its overall deformation is less uniform than the G-type, it holds potential advantages in structural lightweighting and specific strength enhancement.

As shown in Table 5, all three conventional structures exhibit typical stress concentration and gradient distribution characteristics after integrating the aforementioned support rod elements. The D-type unit cell demonstrates significant stress concentration (red to orange regions) at rod connections and boundary areas, indicating heightened stress sensitivity at nodes that may serve as initiation points for plastic deformation or failure. The G-type unit cell exhibits relatively uniform stress distribution, with high-stress zones concentrated along primary load-bearing paths, demonstrating favourable mechanical synergy and load-transfer efficiency. The I-WP unit cell presents intermediate stress levels (yellow to green) with a gentler stress gradient, indicating a well-balanced trade-off between structural stiffness and ductility.

**Table 5:** Static analysis of conventional crystal cell fusion rods.

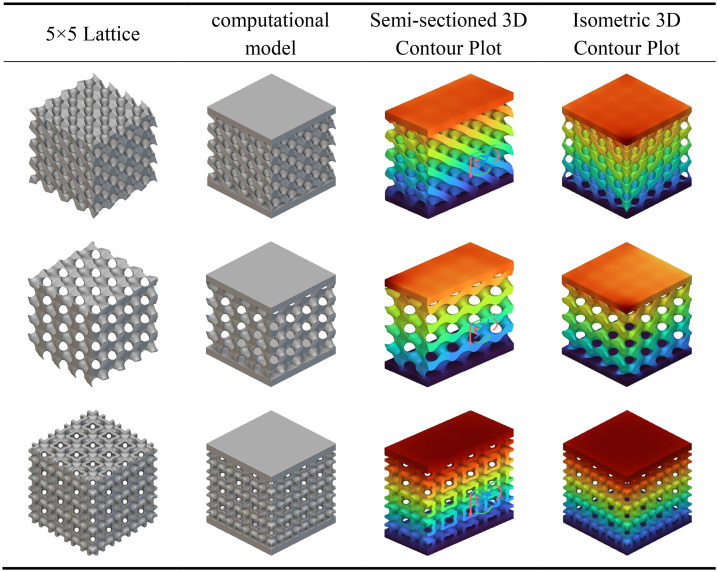

Under identical loading conditions, the differing mechanical behaviours of the three unit cells primarily stem from their inherent capacity for internal stress redistribution, determined by their topological configurations. Cloud diagram results indicate that G-type and I-WP-type structures demonstrate superior performance in uniform load-bearing and stress dispersion, exhibiting potential as lightweight, high-strength materials.

The static analysis results for the transitional fusion crystal cells shown in Table 6 indicate that under boundary conditions of bottom-fixed constraints and a 1000 N vertical load applied at the top, all three fusion crystal cell types (Type D-G, Type G-I-WP, Type D-I-WP) exhibit superior mechanical properties and stress distribution characteristics compared to single configurations.

**Table 6:** Static analysis of transition fusion crystal cells.

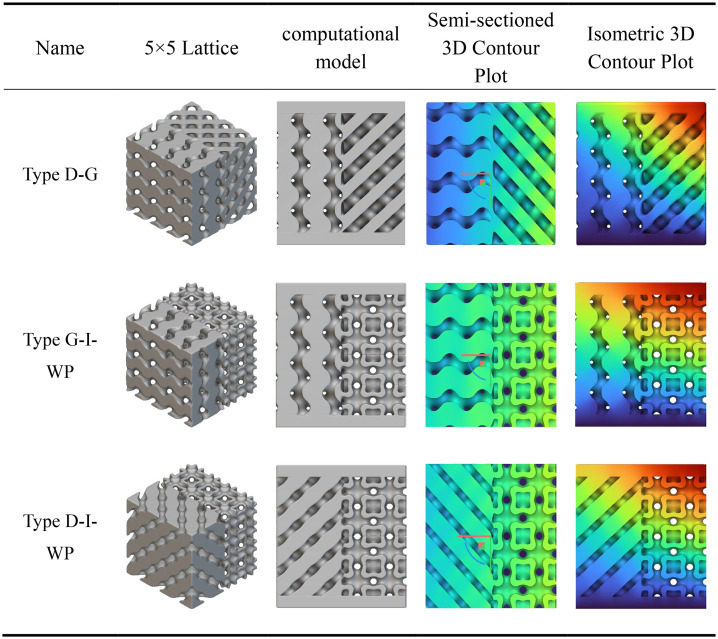

The Type D-G unit cell significantly mitigates the inherent nodal stress concentration inherent to Type D structures by integrating the high rigidity of Type D with the uniform load transfer characteristics of Type G. Stress distribution plots reveal a more dispersed distribution of high-stress zones (red to orange), alongside an expanded area of transitional zones (yellow to green), indicating an optimised load transfer pathway. The Type G-I-WP structure exhibits the smoothest stress gradient transition, confirming the excellent topological compatibility and deformation coordination between the G-type and I-WP-type. This effectively prevents localised stress discontinuities. The Type D-I-WP configuration, following integration, balances the structural rigidity of the D-type with the ductile characteristics of the I-WP-type. Its uniform colour distribution in the stress contour map, devoid of pronounced stress concentrations, demonstrates enhanced load-bearing robustness. Compared to single-type unit cells, the hybrid structure achieves complementary mechanical properties through heterogeneous topological coupling. This not only mitigates stress concentration issues but also enhances the overall structure’s energy absorption efficiency and damage tolerance.

Analysis of the longitudinal displacement contour plots for each TPMS unit cell type during static compression, as shown in Table 7, reveals the following under identical boundary conditions (fixed base, axial compression at top): all unit cells exhibit distinct longitudinal displacement gradients throughout the compression process, with significant variations observed across different topological configurations during the pre-compression, mid-compression, and full-compression stages. Within a single configuration, the Gyroid (G-type) unit cell exhibits the most uniform displacement distribution, characterised by a continuous gradient of colour transitions, demonstrating excellent deformation coordination. Conversely, the Diamond (D-type) unit cell displays more pronounced localised deformation concentration, particularly with larger displacement gradients in the nodal regions.

**Table 7:** Compression longitudinal displacement 3D contour plot.

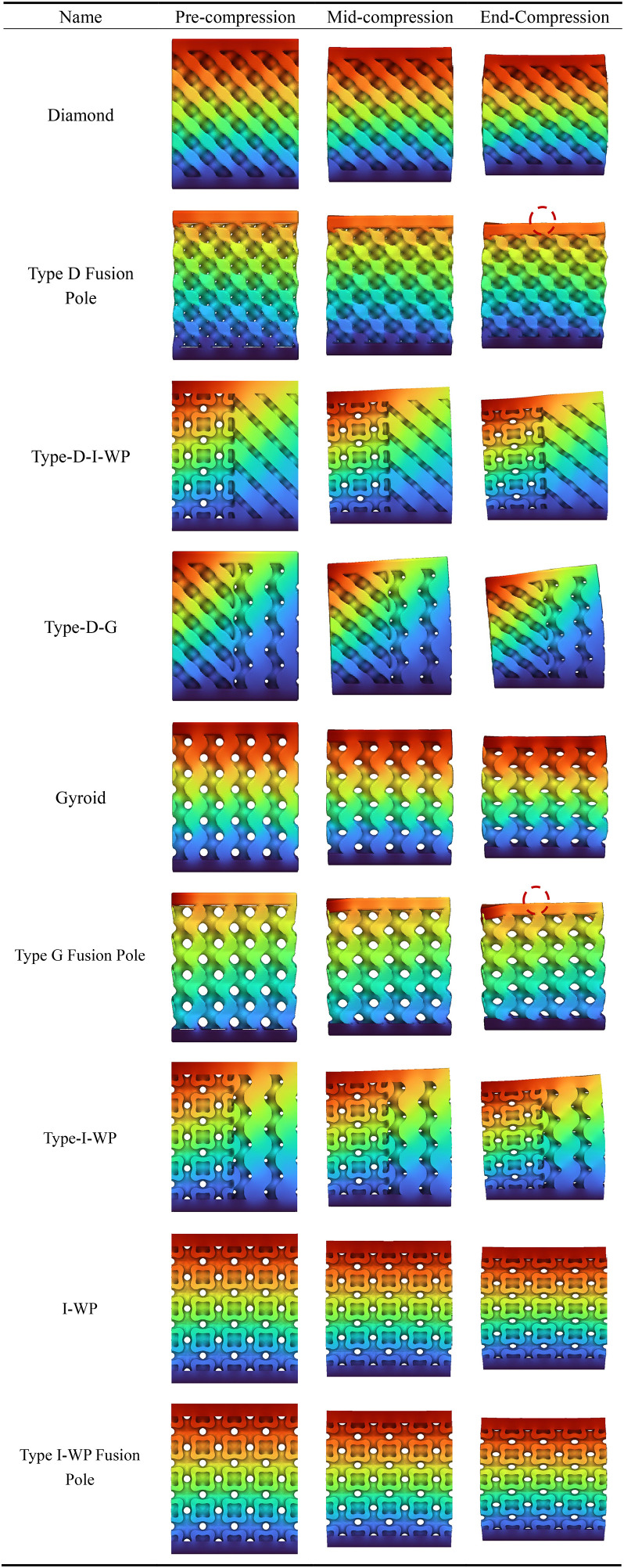

Fused unit cells exhibit deformation characteristics intermediate between their parent structures. The Type D-G (D-G fusion) unit cell partially integrates the high stiffness of Type D with the uniform deformation capability of Type G. Displacement distribution in the central region is more continuous than in pure Type D cells, though mild non-uniformity persists (as indicated by the red circle in the Fig); Type D Fusion Pole exhibits displacement discontinuities at the pole-plane junctions (as indicated by the red circle in the Fig), reflecting deformation mismatch between different topological elements.

The I-WP type and its fusion configuration demonstrate outstanding mechanical properties. The Type I-WP Fusion Pole demonstrated the most uniform longitudinal displacement distribution across all three compression stages, exhibiting smooth and continuous colour transitions without discernible stress concentration phenomena. This characteristic indicates that the configuration achieves excellent deformation coordination at the pole-surface fusion interface, effectively mitigating the adverse effects of localised stiffness discontinuities. Consequently, it exhibits outstanding structural support capability and deformation stability.

**Fig 5:**
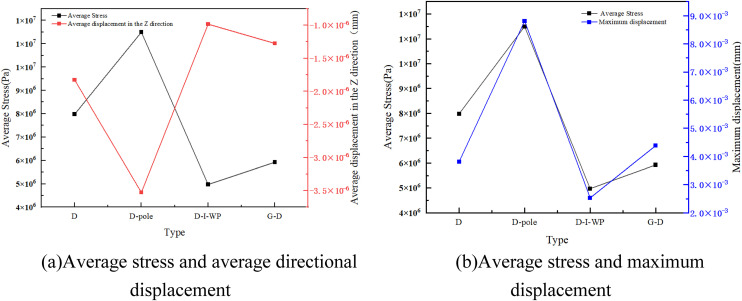
Static Data for Type D. Fig 5 shows the static analysis data of the D-shaped three-periodic minimal surface.

**Fig 6:**
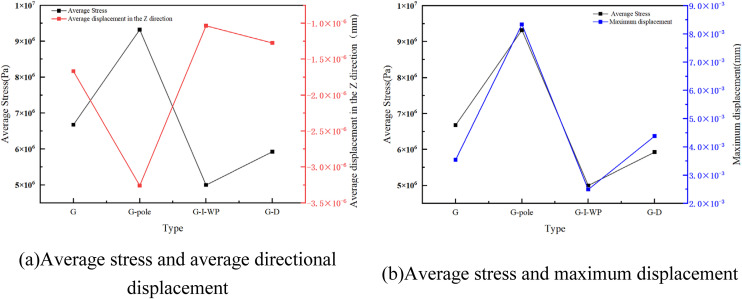
Static Data for Type G. Fig 6 shows the static analysis data of the G-shaped three-periodic minimal surface.

**Fig 7:**
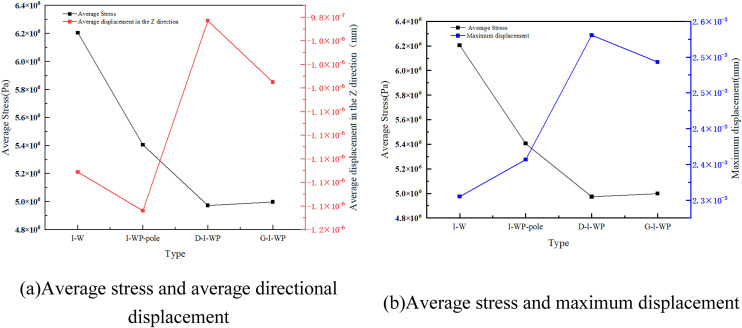
Static Data for Type I-WP. Fig 7 shows the static analysis data of the three-periodic minimal surface of the I-WP configuration.

As illustrated by the results in Figs 5–7, the static data diagram, this study analysed the mechanical response of the TPMS unit cell under compressive loading. It was found that in the D-type and its derivative configurations (Fig 3a), the D-pole fusion rod unit exhibits peak mean stress (approximately 1 × 10⁵ Pa), yet displays significant Z-direction displacement fluctuations (approximately −3.5 × 10 ⁻ ³ mm). This indicates that while rod-surface fusion enhances stiffness, it exacerbates interface deformation mismatch; The D-I-WP configuration exhibited relatively balanced average stress (approximately 7.5 × 10⁴ Pa) and displacement (approximately −2.0 × 10 ⁻ ³ mm), demonstrating the stress distribution improvement effect of heterogeneous topological fusion.

In the G-type configuration (Fig 6b), the G-pole exhibits the highest average stress (nearly 1 × 10⁹ Pa) and the smallest displacement (approximately 2 × 10 ⁻ ³ mm), indicating that the introduction of pole elements significantly enhances stiffness but may compromise deformation capacity; The G-D and G-I-WP configurations achieve a favourable balance between stress levels (approximately 5–7 × 10⁸ Pa) and displacement (approximately 5–7 × 10 ⁻ ³ mm), confirming the advantages of multi-topology fusion in synergistic load-bearing.

In the I-WP configuration depicted in Fig 7, the average stress within the I-WP-pole (i.e., I-WP Fusion Pole) is maintained at a relatively high level (approximately 5.4 × 10⁸ Pa), whilst the maximum displacement in the Z-direction increases moderately (approximately 2.6 × 10 ⁻ ³ m). These data characteristics align with the uniform deformation and absence of stress concentration observed in the contour plots, quantitatively demonstrating the configuration’s comprehensive superiority in structural support capability and deformation stability.

### 3.2. Thermal load analysis

As depicted in the conventional configuration heat transfer contour map shown in Table 8, all three unit cell structures exhibit a characteristic temperature gradient distribution propagating from the heat source towards the apex. The colour gradient transitions gradually from orange-red at the base (high-temperature zone) to blue-violet at the apex (low-temperature zone), reflecting the quasi-isotropic thermal conductivity properties inherent to the porous structure.

**Table 8:** Analysis of heat transfer in traditional unit cells.

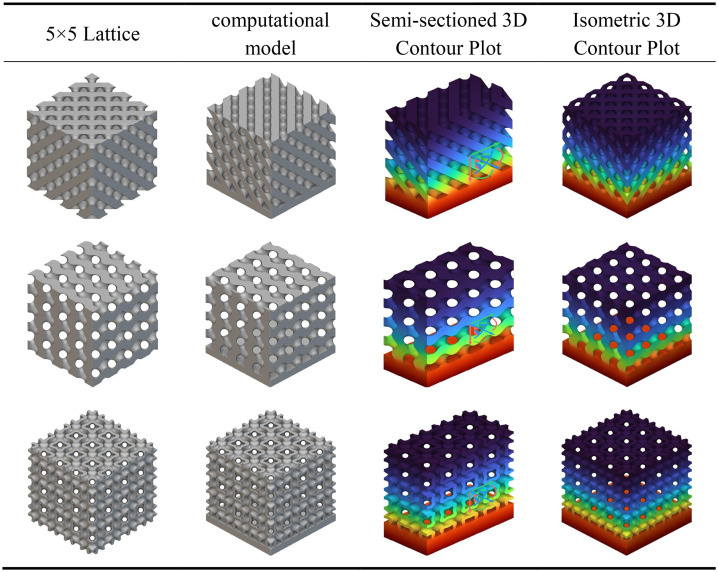

The temperature gradient variation in the D-type unit cell is notably pronounced, with high-temperature regions concentrated at the base and near the pore walls, while the top exhibits lower temperatures. This indicates locally constrained thermal conduction pathways and relatively high thermal resistance. The G-type unit cell demonstrates a more uniform temperature distribution, characterised by smooth colour transitions in the contour plot. Its high-temperature zones extend significantly upwards along the curved surface structure, suggesting that the topological properties of its continuous surface facilitate the formation of efficient thermal flow channels. The temperature field distribution of the I-WP unit cell lies between these two extremes, exhibiting both a degree of gradient smoothness and thermal expansion capability. A noticeable lateral heat diffusion is observable in the central region, reflecting the modulating effect of complex interconnected channels on heat transfer.

According to the heat transfer analysis, contour plots for the conventional crystal cell fusion rod structures shown in Table 9, all three crystal cell fusion rod configurations exhibit a typical temperature gradient transmission from the high-temperature zone at the base (red) to the low-temperature zone at the top (blue). The D-type fusion rod structure displays a steeper temperature gradient variation, with high-temperature regions concentrated at the base and rod-surface connection areas, while the top temperature remains relatively low. This indicates significant constraints in its heat conduction pathways and substantial interfacial thermal resistance. The G-type fusion rod exhibits a more uniform temperature distribution, with smoother colour transitions in the contour plot and a broader upward extension of the heat-affected zone. This demonstrates that the synergistic interaction between its continuous surface and rod elements effectively enhances heat flux transfer efficiency. The I-WP-type fusion rod structure displays a relatively balanced temperature field distribution, combining both longitudinal conduction and lateral diffusion capabilities. Extensive thermal expansion is observable in the central region, highlighting the advantages of porous interconnected structures in thermal management. Under identical thermal boundary conditions, the G-type fusion rod exhibits optimal thermal conductivity and temperature uniformity, with its topological structure significantly reducing thermal resistance. The I-WP type ranks second, while the D-type fusion rod demonstrates relatively weaker thermal management performance due to structural discontinuities at interfaces and localised thermal accumulation phenomena.

**Table 9:** Heat transfer analysis of conventional crystal cell fusion rods.

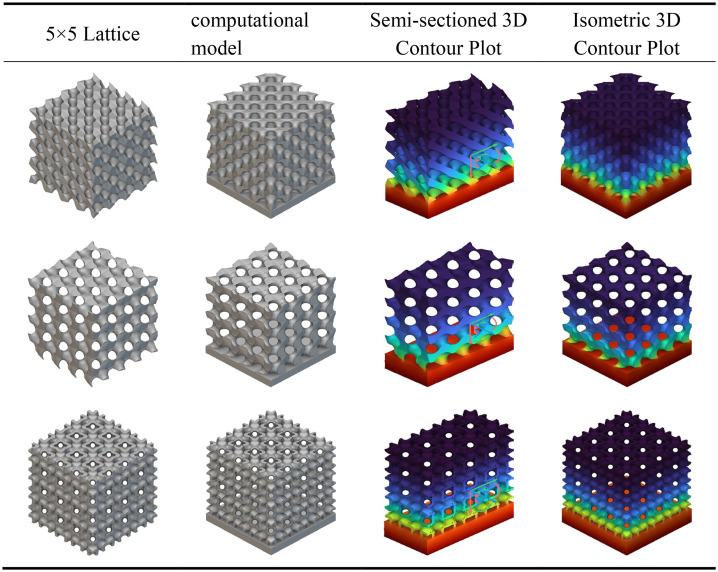

According to the heat transfer analysis results for the transition fusion crystal cells shown in Table 10, all three types exhibit temperature gradient transmission from the high-temperature zone at the base to the low-temperature zone at the top. However, their thermal distribution characteristics show significant improvement compared to the previously mentioned single and rod fusion structures. The G-D fusion structure exhibits the most gradual temperature gradient, with the colour map displaying a continuous transition from top to bottom. The extensive diffusion range of the high-temperature zone indicates that the topological coupling between G and D types effectively enhances thermal conduction uniformity and efficiency. The G-I-WP structure combines favourable vertical thermal conductivity with lateral diffusion capability, revealing pronounced heat flow expansion in the central region, demonstrating the synergistic heat transfer advantages of the porous composite structure. Type D-I-WP exhibits mild thermal accumulation near the bottom heat source, yet demonstrates significant improvement over pure D-type or D-type fusion rod structures, with a more balanced overall temperature distribution.

**Table 10:** Heat transfer analysis of transition fusion crystal cells.

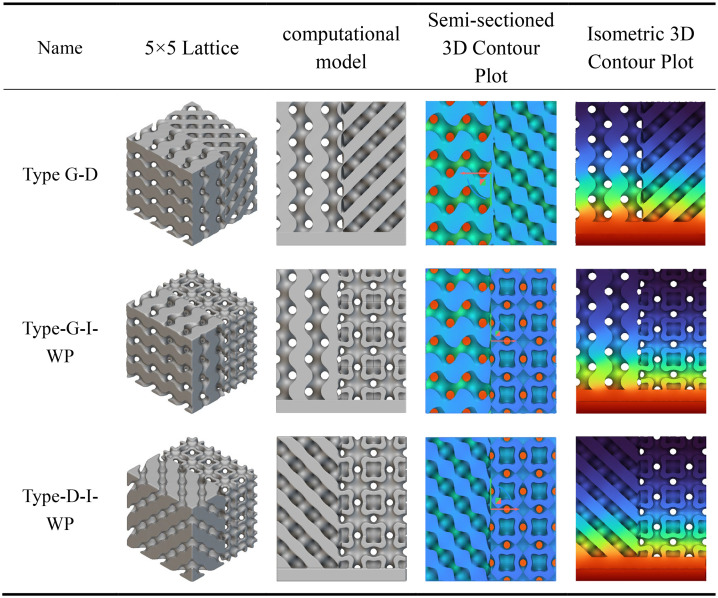

Compared to the single and rod-integrated structures described above, the transition fusion unit cell effectively overcomes interfacial thermal resistance issues through heterogeneous topological coupling, significantly enhancing overall thermal management performance. Among these, Type G-D and Type G-I-WP demonstrate particularly outstanding characteristics. Their high thermal diffusion capacity and uniform temperature distribution properties hold significant application potential in lightweight thermal control structure design.

This paper primarily considers the thermal conduction behaviour within the solid framework of TPMS in its thermal analysis. A heat transfer model based on Fourier’s law is employed to evaluate the internal temperature distribution and equivalent thermal resistance of the structure. Within this modelling framework, fluid flow within the pores and the convective heat transfer effects it induces are excluded from the analysis. Correspondingly, flow-related performance metrics such as permeability and pressure drop are not discussed herein.

This approach constitutes a modelling assumption made in accordance with the research objectives and analytical level of this study. The focus lies on investigating how the topological configuration of TPMS and its transition fusion methods influence the continuity of solid thermal conduction pathways and the structural load-bearing capacity, aiming to reveal the intrinsic relationship between variations in structural parameters and the solid’s thermal and mechanical responses. Within this research context, introducing fluid-structure interaction or pore-scale flow models would substantially increase computational complexity and potentially compromise the analysis and comparison of topological effects themselves.

In thermally managed metamaterials where fluid cooling or seepage heat transfer predominate, permeability, pressure drop, and convective heat transfer performance often hold more direct engineering significance. Such problems typically necessitate systematic investigation through fluid-structure interaction or multiscale numerical methods, predicated upon well-defined fluid operating conditions and boundary constraints. While relevant analyses extend beyond the scope of this paper, subsequent work will build upon existing structural design and homogenisation frameworks. This will involve introducing pore-scale flow simulations to conduct supplementary studies on the seepage and convective heat transfer performance of TPMS transition-blended structures.

### 3.3. Homogenisation analysis

It should be noted that the TPMS transition-blended structures investigated herein do not strictly satisfy the condition of complete isotropy. Although certain fundamental TPMS configurations may exhibit approximately isotropic mechanical responses under the idealised assumption of infinite periodicity, the introduction of configuration transitions, finite unit cell dimensions, and specific loading and boundary conditions inevitably results in a degree of directional dependence in their isotropic mechanical properties. In light of this understanding, the revised manuscript has uniformly amended the original term ‘isotropic’ to ‘quasi-isotropic’ to more accurately reflect the structure’s actual macroscale mechanical characteristics.

**Fig 8:**
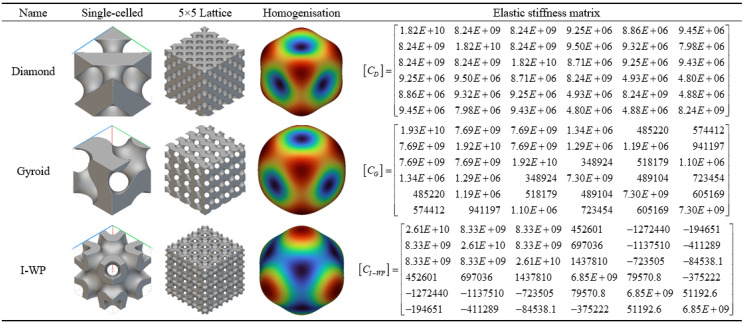
Conventional Homogenisation Analysis. Fig 8 shows the homogeneous normalization model of the traditional three-periodic minimum surface structure.

As illustrated in Fig 8, the disparity between the elastic modulus and shear modulus components along different principal directions primarily stems from the spatially non-uniform distribution of structural geometry during the transition fusion process. The transition function introduces continuous yet non-symmetrical topological evolution along specific directions, resulting in distinct effective load-bearing pathways and stress transfer mechanisms within the unit cell across different orientations. This leads to the homogenised equivalent stiffness matrix exhibiting weak anisotropic characteristics. Such phenomena are commonplace in gradient structures and non-periodic metamaterials, and do not indicate anomalies in structural design or numerical implementation.

Furthermore, negative shear stiffness terms observed under certain operating conditions require interpretation within the physical context of homogenisation calculations. This phenomenon does not indicate macroscopic mechanical instability or violation of positive energy in the material, but rather stems from contributions to macroscopic shear response by local rotation, bending, or geometric rearrangement effects within the unit cell under specific shear loading patterns. When shear strain energy is predominantly governed by structural rearrangement rather than pure shear deformation, the equivalent shear stiffness component may exhibit numerically small negative values or near-zero results. Similar phenomena have been reported in homogenisation studies of porous metamaterials and buckling-dominated lattice structures.

Subsequent analysis in this paper focuses on the overall symmetry and positive definiteness of the equivalent stiffness matrix, rather than the sign variation of individual shear components. Verification confirms that the equivalent stiffness matrices for all configurations satisfy energy stability conditions, with structures exhibiting no macroscopic instability within the considered load range. Consequently, negative shear stiffness terms are interpreted herein as reflecting structural deformation mechanisms rather than numerical errors or model deficiencies. Based on this understanding, interpretations and conclusions regarding relevant results have been revised accordingly to ensure the accuracy and rigour of physical meaning.

Based on the homogenisation analysis results of the conventional TPMS unit cell shown in Fig 8, the calculated equivalent elastic stiffness matrix indicates that all three unit cell structures exhibit pronounced quasi-isotropic mechanical behaviour. Within the stiffness matrix of the D-type unit cell, the diagonal elements C₁₁, C₂₂, and C₃₃ exhibit high values (approximately 1.82E + 10 Pa), while the off-diagonal coupling terms C₁₂ and C₁₃ remain at the order of 8.24E + 09 Pa. This indicates a high axial stiffness coupled with relatively balanced lateral coupling stiffness. However, shear stiffness terms (such as C₄₄, C₅₅, C₆₆) exhibit significantly reduced values (approximately 8.24E + 09 Pa), reflecting the structure’s weaker response under shear loading. In contrast, the stiffness matrix of the G-type unit cell exhibits more uniform mechanical properties, with principal stiffness components C₁₁, C₂₂, and C₃₃ approximately 1.92–1.93 × 10¹⁰ Pa, and consistent transverse coupling terms (around 7.69 × 10⁹ Pa). Notably, its off-diagonal and shear stiffness values are markedly lower than those of the D-type (e.g., C₄₄ at approximately 7.30E + 09 Pa), indicating that this structure maintains high rigidity while exhibiting superior deformation coordination. The I-WP-type unit cell displays unique stiffness characteristics: its principal stiffness components are significantly enhanced (C₁₁, C₂₂, C₃₃ reaching 2.61E + 10 Pa), yet coupling terms like C₁₂ are only 8.33E + 09 Pa, with shear stiffness terms exhibiting negative values (e.g., C₄₄ and C₅₅ at approximately 6.85E + 09 Pa). This suggests the structure may exhibit atypical deformation behaviour under complex stress conditions.

The fourth and fifth columns of the homogenised cloud maps further elucidate the deformation mechanisms within each unit cell: the D-type cloud map exhibits concentrated colour distribution, indicating a relatively localised strain energy distribution; the G-type cloud map displays uniform colour transitions, confirming its excellent mechanical coordination; the I-WP-type cloud map presents a gradient variation, corresponding to the asymmetric terms within its stiffness matrix.

**Fig 9:**
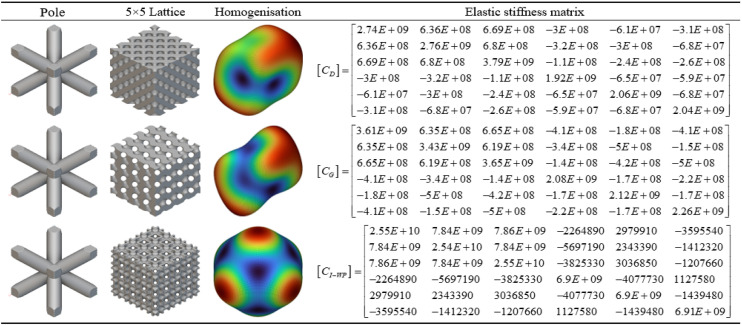
Homogenisation Analysis of the Fusion Bar Configuration. Fig 9 shows the mean normalization model of the three-periodic minimal surface fusion rod unit configuration.

Based on the homogenisation analysis results for the fusion rod configuration shown in Fig 9, the homogenised calculations demonstrate that the fusion rod structure significantly enhances the stiffness performance of the original TPMS topology. Within the elastic stiffness matrix of the D-type fusion rod, principal stiffness components C₁₁, C₂₂, and C₃₃ all exhibit substantial increases (generally reaching the 10⁹–10¹⁰ Pa magnitude), while non-diagonal coupling terms such as C₁₂ and C₁₃ concurrently strengthen. This indicates the rod elements effectively enhance the structure’s axial and lateral load-bearing efficiency. The G-type fusion rod maintains high principal stiffness (e.g., C₃₃ ≈ 1.92E + 10 Pa) while exhibiting superior shear stiffness elements (C₄₄, C₅₅, etc.) compared to conventional G-type structures, demonstrating the synergistic toughening effect of the ‘plate-rod’ interaction. The stiffness matrix of the I-WP fusion rod exhibits further optimised values across multiple parameters, with notably enhanced coupling terms such as C₁₃ and C₂₃. This demonstrates superior stress transfer and deformation resistance under complex multi-directional loading conditions.

The homogenisation cloud diagram reveals that the strain energy distribution across the three types of composite rod structures is more uniform, exhibiting a smoother colour gradient. Furthermore, the high-stiffness regions (red to yellow) have expanded, providing further evidence that the introduction of rod elements effectively enhances and homogenises mechanical properties

**Fig 10:**
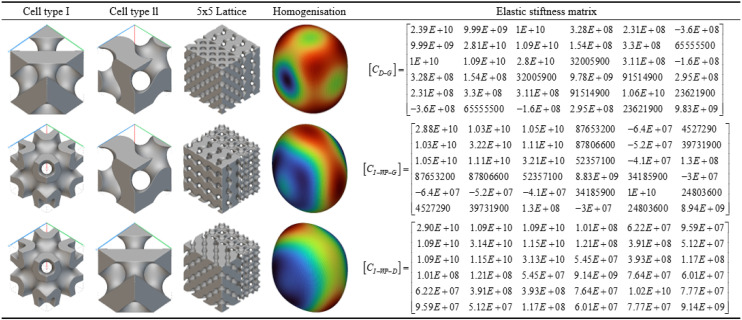
Transitional Fusion Homogenisation Analysis. Fig 10 shows the three-periodic minimal surface multi-configuration transitional fusion homogeneous normalization model.

According to the multiphase TPMS Lattices homogenisation analysis results shown in Fig 10, this configuration demonstrates significant structural synergistic advantages in terms of equivalent elastic properties. Homogenisation calculations indicate that the three multiphase TPMS Lattices structures—D-G, I-πg-G, and I-πp-D—achieve systematic enhancement of the elastic stiffness matrix through heterogeneous topological coupling: Their principal stiffness components (e.g., C₁₁, C₂₂, C₃₃) generally attain values of 10¹⁰ Pa, while off-diagonal coupling terms (e.g., C₁₂, C₁₃) are markedly strengthened, reflecting an excellent synergistic load-bearing mechanism under multidimensional stress conditions. Homogenised contour plots further reveal highly uniform strain energy distributions across all three structures, with expanded high-stiffness regions (warm colour tones) and smooth colour transitions. This confirms the critical role of heterogeneous interfaces in promoting stress diffusion and suppressing localised deformation. By integrating multiple topological advantages, multiphase TPMS Lattices design overcomes the performance limitations of single configurations, establishing a new paradigm for the targeted design and cross-scale regulation of high-performance lightweight porous structures.

In the implicit function-based TPMS transition blending modelling process, the transition steepness coefficient k serves as the key parameter governing the rate of geometric change between different topological configurations. Its physical significance may be understood as the smoothness of the topological transition region. A smaller k value corresponds to a more gradual structural transition, whereas a larger k value induces rapid spatial switching of the topological morphology, thereby generating a pronounced geometric gradient in localised regions. Consequently, it is necessary to examine the impact of parameter k variations on structural mechanical properties such as strength.

**Fig 11:**
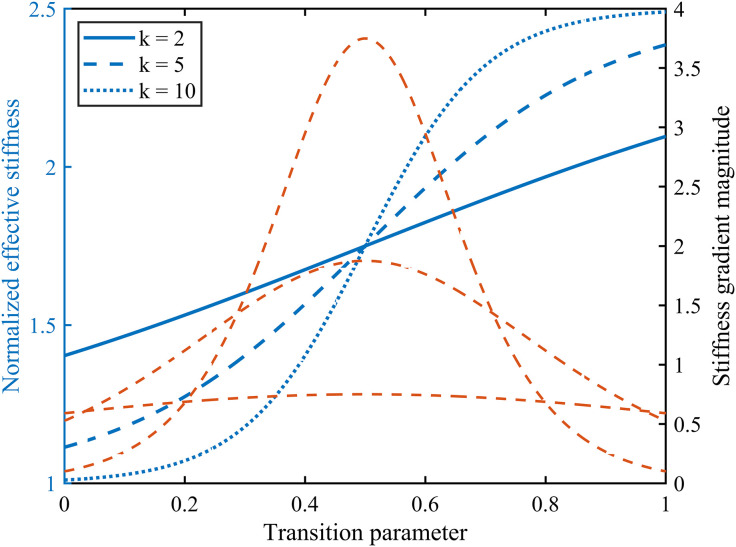
Sensitivity analysis of the steepness coefficient k of the transition. Fig 11 shows the sensitivity analysis data graphs when K is 2, 5, and 10.

As illustrated in Fig 11, this study conducted a comparative analysis of transition-blended structures under varying k values while maintaining constant unit cell dimensions, relative density, and transition start/end positions. Results indicate that as k increases, the transition zones between different topological regions within the structure progressively contract, with the distribution of equivalent elastic stiffness exhibiting a shift from smooth variation towards segmented characteristics. Within a narrow k range, the overall structural stiffness exhibits relatively continuous variation with the transition parameter, accompanied by a moderate anisotropic response. This facilitates a coordinated transition in mechanical properties. However, as k increases further, local stiffness gradients become markedly enhanced. Although the overall equivalent stiffness level changes only marginally, the disparity between stiffness components in different principal directions amplifies.

Within the parameter range examined herein, variations in k do not induce abrupt changes in the order of magnitude of the equivalent elastic modulus. Its influence is primarily manifested in the trend of stiffness distribution and mechanical coordination. Consequently, the transition steepness coefficient k is regarded in this study as a design parameter for regulating the smoothness of structural performance, rather than a dominant factor determining the upper limit of macroscopic mechanical properties. Based on the foregoing analysis, subsequent inverse design and performance mapping in this paper employ moderate k values as representative, thereby achieving a balance between structural continuity and performance differentiation.

### 3.4. Inverse engineering examples

Mechanical Objective: Designed to meet requirements for traditional medical orthoses, the target range for axial equivalent stiffness is 185–190 GP.

Thermal Objective: Excellent effective thermal conductivity, manifested as uniform temperature distribution in thermal analysis with no significant hotspots (qualitative assessment based on heat transfer analysis results in Section 3.3 and Table 10).

Preliminary Screening: The single D-type stiffness slightly undershot the lower target limit, the single G-type stiffness slightly exceeded the upper target limit, while the single I-WP-type stiffness proved excessively high. Consequently, focus shifted to transitional hybrid configurations enabling stiffness ‘fine-tuning’.

Candidate Configuration Determination: The equivalent stiffness of the D-G hybrid configuration near the target range (approximately 188 GPa) precisely falls within the 185–190 GPa target interval. Concurrently, its thermal analysis results (Table 10) demonstrate uniform temperature field distribution, satisfying the thermal objective. Consequently, the D-G hybrid configuration is selected as the optimal candidate.

Determining transition parameters: Based on sensitivity analysis of the transition steepness coefficient, K = 2 and $\gamma_{\text{0}}=0$ (i.e., 50% blending ratio) were selected. This parameter combination ensures structural rigidity requirements are met while providing smooth stress transitions and favourable deformation coordination.

By employing the D-G hybrid implicit function (Equation 5) from Section 2.2 and substituting the determined parameters K = 2 and into Equation 5 above, the D-G hybrid structural geometry satisfying the target performance requirements can be directly generated. This structure exhibits a smooth topological transition from G-type to D-type, with schematic representations of its geometric configurations shown in Figs 1b and 2.

**Fig 12:**
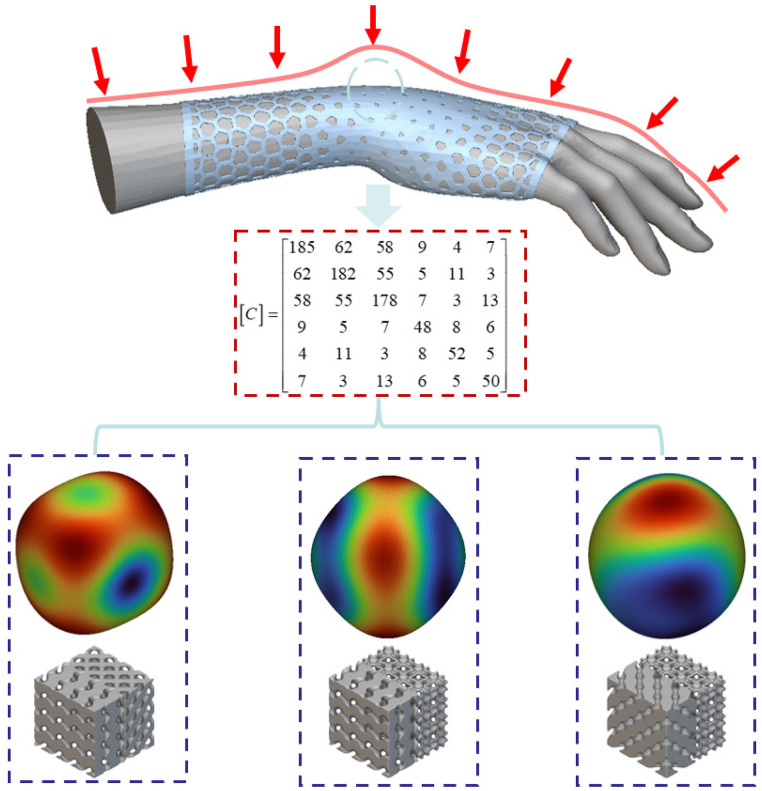
Homogenised Element Derived from Inverse Stiffness Matrix. Fig 12 shows the schematic diagram of the application principle of the homogenized unit cell derived from the inverse stiffness matrix.

The schematic diagram of the principle is shown in Fig 12. Based on the preceding discussion, the elastic stiffness matrix (unit: GPa) conforming to standard medical right-hand braces is employed as input conditions. Following the aforementioned approach, the corresponding homogenised element is derived through reverse calculation, ultimately enabling the inference of its potential fused unit cell structure.

## 4. Conclusion

1. This study constructs functions suitable for TPMS transitional fusion by capturing periodically infinite, continuous, and smooth surfaces through surface boundary capture techniques, based on the fundamental configuration of tri-periodic minimal surfaces. It establishes new tri-periodic minimal surface configurations—D, G, and I-WP types fused with pole elements, as well as mutually smooth-fused variants. By synthesising the advantages of these configurations, novel metamaterials with both excellent load-bearing and heat transfer capabilities were designed. Through pole unit fusion (e.g., I-WP Fusion Pole) and heterogeneous topological transitions (e.g., Type G-D), the stress distribution uniformity, stiffness, and thermal conductivity efficiency of TPMS structures were significantly optimised, addressing the performance shortcomings of single configurations in either load-bearing or heat transfer.
2. This study presents a method for constructing implicit functions for each corresponding new configuration by integrating the corresponding transition fusion function. Through this implicit function formula, multiple new configurations of metamaterials required for fusion can be directly constructed.
3. Ultimately, through homogenisation analysis of each novel configuration, this study obtained the mechanical vector data for each new structure. The homogenisation analysis demonstrated that the elastic stiffness matrix of the integrated configurations (including principal components such as C₁₁, C₃₃, and coupling terms) exhibited systematic enhancement, with a more uniform distribution of strain energy. validating the controllable correlation between structure and performance. Combined with the implicit function formulation, this configuration and design methodology establish a novel theoretical approach for reverse-engineering mechanically tunable metamaterials tailored to specific requirements.

### Nomenclature

**Table:** 

T	temperature (°C)
t	time (s)
𝐟	force (TaN)
p	pressure (Pa)
k	Transition Steepness Coefficient
ϕ(γ)	Hidden function symbol
ω(γ)	Weighting function
C	Matrix symbol
***Greek symbols***	
γ	Transition control variable

Symbol Explanation Table.

## Supporting information

S1 Table The high-definition vector table of the first part of the article. (ZIP)

S2 Table The high-definition vector table of the second part of the article. (ZIP)

S3 Table The high-definition vector table of the third part of the article. (ZIP)

S4 Table The high-definition vector table of the fourth part of the article. (ZIP)

S5 Table The high-definition vector table of the fifth part of the article. (ZIP)

S6 Table The high-definition vector table of the sixth part of the article. (ZIP)
